# Scientific achievements and reflections after 20 years of vector biology and control research at the Pu Teuy mosquito field research station, Thailand

**DOI:** 10.1186/s12936-022-04061-5

**Published:** 2022-02-14

**Authors:** Patcharawan Sirisopa, Chutipong Sukkanon, Michael J. Bangs, Sutkhet Nakasathien, Jeffrey Hii, John P. Grieco, Nicole L. Achee, Sylvie Manguin, Theeraphap Chareonviriyaphap

**Affiliations:** 1 Department of Entomology, Faculty of Agriculture, Kasetsart University, Bangkok, 10900 Thailand; 2 Department of Medical Technology, School of Allied Health Sciences, Walailak University, Nakhon Si Thammarat, 80160 Thailand; 3 Department of Agronomy, Faculty of Agriculture, Kasetsart University, Bangkok, 10900 Thailand; 4 College of Public Health, Medical and Veterinary Sciences, James Cook University, North Queensland, QLD 4810 Australia; 5 Department of Biological Sciences, Eck Institute for Global Health, University of Notre Dame, Notre Dame, IN USA; 6 HSM, Univ. Montpellier, CNRS, IRD, IMT, Montpellier, France

**Keywords:** Semi-field system, Repellent, Experimental hut, Mosquito ecology, Vector-borne diseases, Thailand

## Abstract

Additional vector control tools are needed to supplement current strategies to achieve malaria elimination and control of *Aedes*-borne diseases in many settings in Thailand and the Greater Mekong Sub-region. Within the next decade, the vector control community, Kasetsart University (KU), and the Ministry of Higher Education, Science, Research and Innovation must take full advantage of these tools that combine different active ingredients with different modes of action. Pu Teuy Mosquito Field Research Station (MFRS), Department of Entomology, Faculty of Agriculture, Kasetsart University (KU), Thailand was established in 2001 and has grown into a leading facility for performing high-quality vector biology and control studies and evaluation of public health insecticides that are operationally relevant. Several onsite mosquito research platforms have been established including experimental huts, a 40-m long semi-field screening enclosure, mosquito insectary, field-laboratory, and living quarters for students and researchers. Field research and assessments ranged from ‘basic’ investigations on mosquito biology, taxonomy and genetics to more ‘applied’ studies on responses of mosquitoes to insecticides including repellency, behavioural avoidance and toxicity. In the course of two decades, 51 peer-reviewed articles have been published, and 7 masters and 16 doctoral degrees in Entomology have been awarded to national and international students. Continued support of key national stakeholders will sustain MFRS as a Greater Mekong Subregion centre of excellence and a resource for both insecticide trials and entomological research.

## Background

The long-term effectiveness of current approaches to malaria control such as artemisinin-based combination therapy, indoor residual spraying and insecticide-treated materials are undermined by increasing antiparasitic drug resistance, physiological resistance and behavioural responses of mosquito vectors to insecticides [1, 2]. Controlling *Aedes* species, a cosmo tropical vector of dengue, yellow fever, Chikungunya and Nipah viruses remains difficult due to weak evidence from appropriately designed trials to reach a conclusion about any of the control methods available [3, 4] Consequently, there is an increasing need for new strategies that exploit novel aspects of vector genetics, physiology, behaviour and ecology. These innovations must be drawn from an understanding of vector biology within natural transmission settings if they are to yield rapid, locally appropriate strategies for disease control [5]. For example, the combination of laboratory and confirmatory field studies led to a new paradigm for classifying chemicals used for vector control according to how the chemicals actually function to prevent disease transmission inside houses. It was proposed that the new classification scheme will characterize chemicals on the basis of spatial repellent, contact irritant and toxic actions [6] which partly explains the excito-repellency effect of DDT in reducing human-vector contact field-based experimental hut studies in forested areas of Thailand [7].

Indeed, closer integration of laboratory-based excito-repellency box (ERB) test systems and field experimental hut studies showed the complexity of the impact of insecticides on levels of behavioural responses of *Anopheles* species, and the role of behavioural resistance in reducing the selection pressure and spread of insecticide resistance [8]. Additional studies have shown that the success of ERB assays depends on the procedural ease for introducing and removing female mosquitoes in a semi-field system which is “ideally situated within the natural ecosystem of the target disease vector and exposed to ambient environmental conditions, within which all features necessary for its lifecycle completion are present” [5, 9–11]. These limitations may account for the lack of precise estimates of the value and variability of the repelling effect of any product inhibiting outdoor biting versus its killing and disarming (preventing host-seeking until the next night) effects based on human landing collection data obtained from controlled Semi-Field System (SFS) experiments [12]. Clearly, to assess the effectiveness of candidate tools in an early stage of product development, intermediary testing grounds between the laboratory and field within disease-endemic countries are needed to fight outdoor malaria transmission and *Aedes*-borne viral diseases [13].

It is crucial for vector-borne disease control programmes to enhance the vector biology and control research activities and importantly for policy makers to prioritize evidence-based intervention strategies. Control of vector borne diseases in Thailand, like elsewhere, largely depends on vector management [13]. In this regard, the Department of Entomology, Faculty of Agriculture, KU, Thailand, established the Pu Teuy Mosquito Field Research Station (MFRS) in 2001 located in Kanchanaburi Province with experimental huts, a 40-m long semi-field system (SFS) enclosure, mosquito insectary, field-laboratory, and living quarters for students and researchers. Because the abundance and composition of vectors within the SFS can be known *a priori*, experimental manipulation (either at the time of introduction, or through removal of some target individuals) can produce much more precise estimates of the value and variability of demographic and life-history parameters than would be from direct field observations [5]. Additionally, SFS also enables additional entomological endpoints beyond simply HLC – i.e. mortality, disarming, fecundity that would not be possible in open field trials. MFRS is one of the few research stations in the world that has operational SFS in the Asia–Pacific region while others are mostly located in Sub Saharan African countries, for example in Burkina Faso, Sudan, Kenya, Tanzania and Zambia [5, 14–17]. At ÷the Ifakara Health Institute in southern Tanzania where one of the world’s biggest SFS was established in 2005, several vector biology, ecology, parasite-vector interaction, chemical efficacy studies on *Anopheles arabiensis* and other mosquito vectors have been conducted [5, 18]. Similarly, studies of several African malaria vectors were studied at Mbita Point Field Station in western Kenya [19]. Experimental huts are important alternatives to use of actual human dwellings, because of ethical regulations governing human participants in mosquito behaviour and pesticide studies. The huts are recommended by the World Health Organization (WHO) for assessing the efficacy of indoor residual spraying and insecticide treated nets (ITNs) during the second phase of testing protection potential insecticidal products [20, 21] As the design of the huts varies across different regions, with diversity of *Anopheles* and *Aedes* mosquito behaviours, several Asian design experimental hut studies in Thailand have been described in this paper. Currently there are two sites with SFS, one, MFRS in Kanchanburi Province, and the second AFRIMS field station in Kamphaeng Phet Province [22]. As a strategic resource for conducting vector related research during the last two decades, the MFRS offers excellent opportunity to study the impact of pesticides, repellents and parasite-vector interactions [5].

Under a collaborative agreement between KU and the Thai Military Development Office, the Department of Entomology, Faculty of Agriculture, KU, has maintained MFRS to support entomological research of various insect species of public health importance, especially mosquitoes and dipteran flies. Over the past two decades, a total of 80 undergraduates and 23 postgraduates (7 masters and 16 doctorate students) conducted research projects at MFRS and published 51 peer-reviewed articles in national and international journals. Basic entomological research pertaining to the biology and taxonomy of various insect species, especially mosquitoes, were studied in considerable detail. Various mosquito trapping systems such as, the BG-Sentinel™ (BGS) trap [23] have been developed, and tested utilizing experimental huts constructed in situ. Insecticide efficacy tests such as those conducted to evaluate mosquito behavioural responses using the excito-repellency assay system, and toxicity tests have also been carried out at this field station [24–26]. In addition, several national and international training courses on Malaria Vector Surveillance for Elimination (MVSE), vector identification and vector surveillance by the French Research Institute for Sustainable Development (IRD), and field training on new mosquito surveillance methods supported by the Asia Pacific Malaria Elimination Network (APMEN) have been conducted at this field research station.

The aim of this paper was to compile and consolidate the history and publications on vector biology, vector control and the main research activities conducted at the MFRS over the past 20 years. The research programme is crucial to the development of a district-focused MFRS capable of generating new knowledge and information for public health policy and action. Thus, it was envisioned that the MFRS will become a centre of excellence and innovation in vector control research and development with aspiration of becoming a leading public health field research institute in the Greater Mekong subregion and globally.

### Landscape

**Fig. 1:**
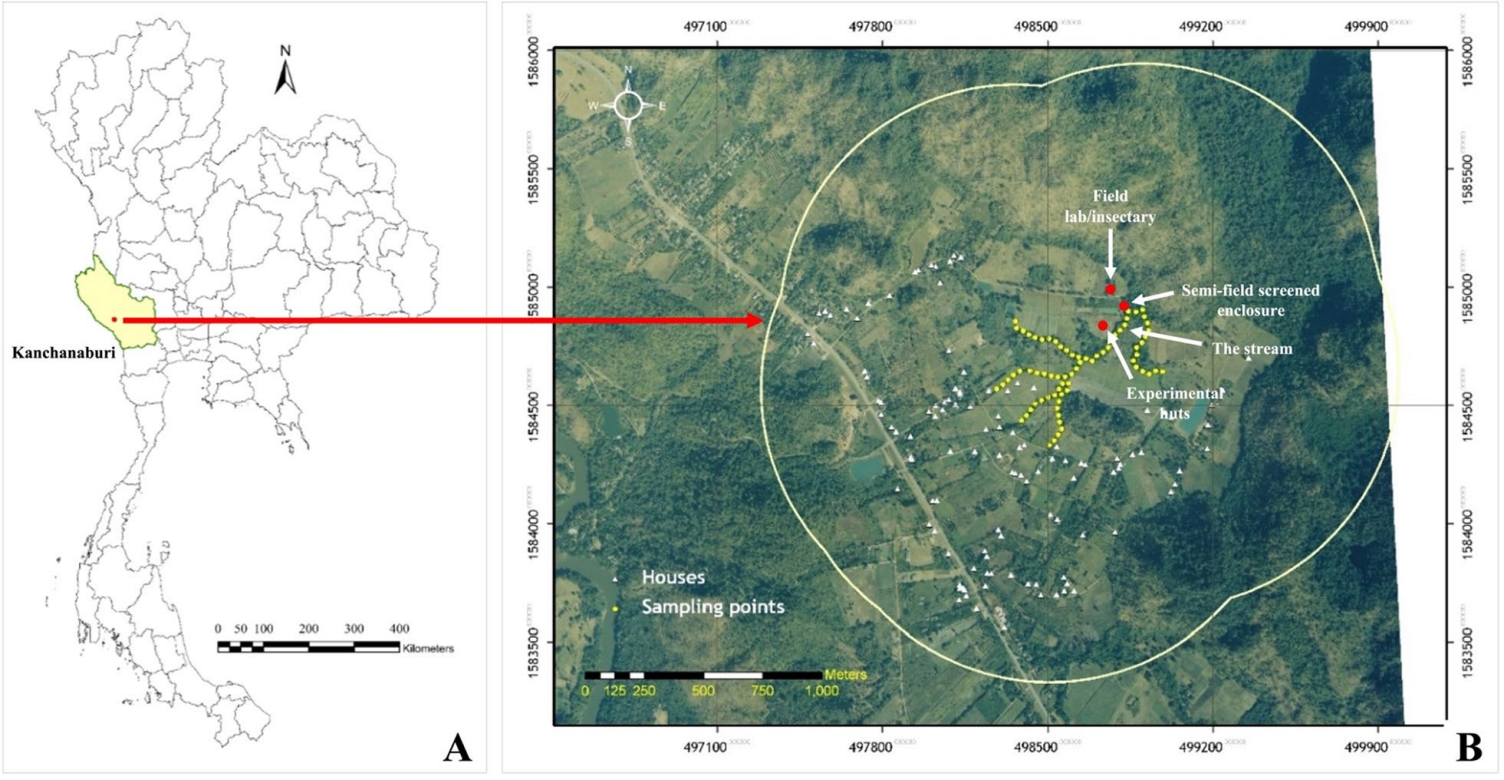
Map of Thailand with **A** Kanchanaburi Province, in western region and **B** satellite map of Pu Teuy Village where Pu Teuy Mosquito Vector Research Station is located, yellow dots represented the stream near the field station

The MFRS is located in an area vegetated with screw pine trees (*Pandanus tectorius*) and perennial underground streams and provided an adequate environment for scientific resource of high academic research for KU. Pu Teuy Village is one of the eleven villages of Ban Ta Sao Sub-district, Sai Yok District, Kanchanaburi Province, western Thailand (14˚ 17́́́ N, 99˚11́ E, 310 m asl) (Fig. 1A). It is located near the Sai Yok Noi Waterfall, a beautiful tourist attraction in the Valley of Khwae Noi River among the Tenasserim hills area. Pu Teuy Village has a population of 939 inhabitants whose major occupation is agriculture [27] and forest activities, principally logging, hunting, forest food gathering and forest protection [28]. The MFRS is situated at the base of a hilly terrain mostly surrounded by thick natural and planted forests, approximately 800 m from the nearest house in the village. The main water body near the site is a narrow effluent stream that flows from the base of the hills under native vegetation [28] (Fig. 1B). The thick forest cover and water body provide a conducive breeding ground for many local mosquito vectors such as *Anopheles minimus*, *Anopheles harrisoni*, *Anopheles dirus*, *Anopheles maculatus*, and *Stomoxys* spp. [29, 30].

Although, human activities in the area have gradually increased such as deforestation, hunting and forest food gathering, the natural environment of Pu Teuy Village has remained relatively intact sustaining its current mosquito species composition [31]. For example, the medically important *An. minimus* complex comprising *An. minimus *sensu stricto (*s.s*.) and *An. harrisoni* is abundant and maintained in a numerical ratio of 1:3 since 2002 [32]. Recent records showed that > 90% of *An. minimus* complex belongs to *An. harrisoni* [33–35].

### Vector-borne diseases status and vector control strategies in Sai Yok District, Kanchanaburi Province

Despite decades of organized vector control efforts, vector-borne diseases remain persistent threats and continue to impose a public health burden to vulnerable populations in receptive areas of Thailand [8]. Among these areas, Kanchanaburi Province is endemic for malaria and dengue, the two most commonly notifiable infections along with a few cases of lymphatic filariasis, Zika, and Chikungunya [4].

**Fig. 2:**
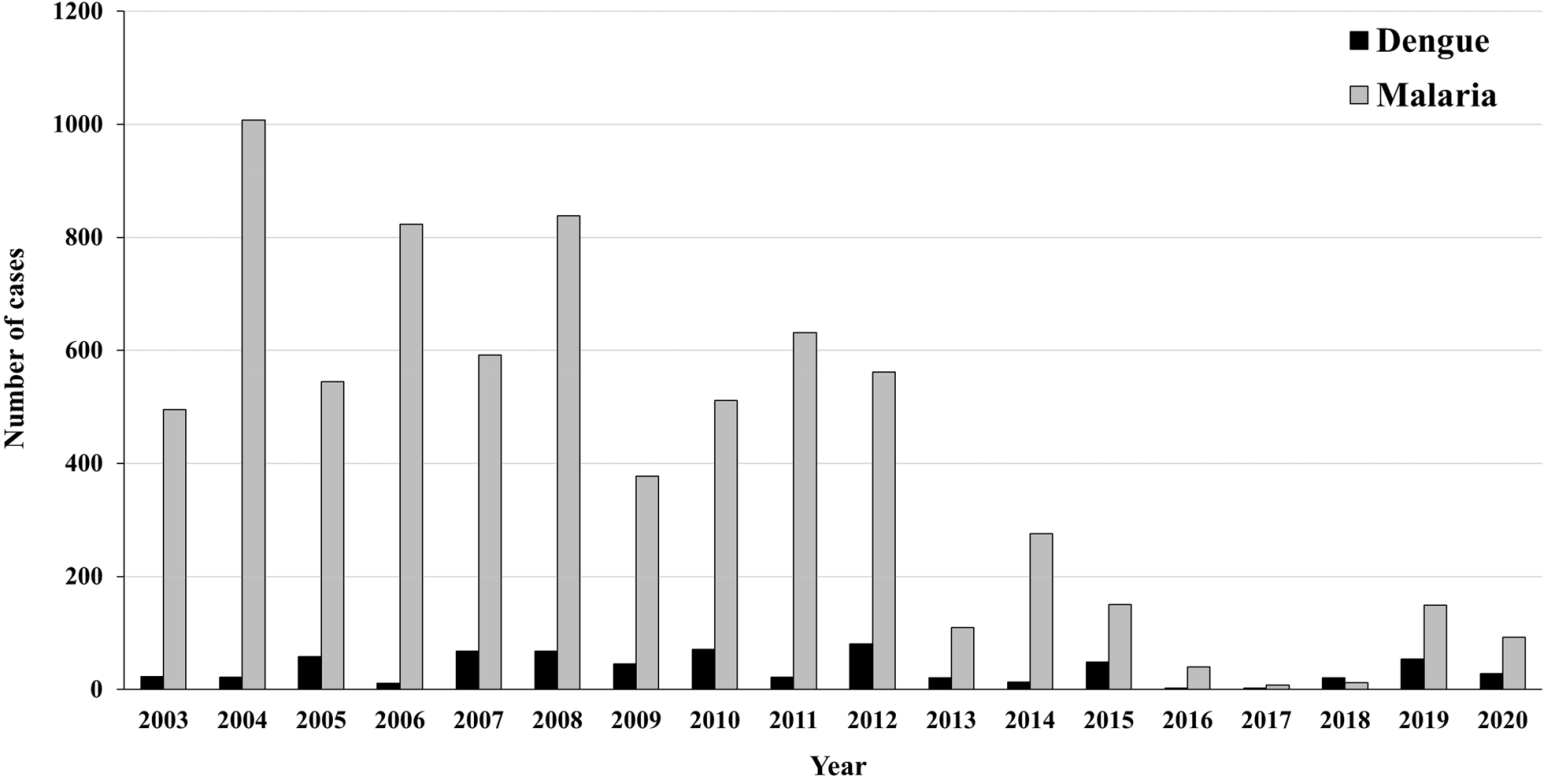
Number of dengue and malaria cases in Sai Yok District, Kanchanaburi Province, from 2003 to 2020 [37]

As one of the districts with a high incidence of vector-borne diseases, Sai Yok District lies in the tropical climate zone conducive for perennial malaria transmission with most cases reported during the wet season (May to November) [36]. Between 2004–2020, the number of malaria cases detected by MOPH in Sai Yok District fell from 1,007 to 92 cases, but this decline was reversed due to a sharp increase from 8 (2017) to 149 cases in 2019 (Fig. 2). In 2020, the annual parasite incidence (API) of malaria in Sai Yok District was 92 cases per 1,000 population [37], and a parasite ratio of 0.98 (*Plasmodium vivax*): 0.02 (*Plasmodium falciparum*). At the same time, dengue incidence increased from 2 in 2004 to 81 cases in 2019; and a drop to 28 cases in 2020 [37]. As of 2021, Sai Yok District was identified as a dengue risk area [4].

The ecology and behaviour of mosquito vector populations are key determinants for selecting the most appropriate and efficacious intervention methods for the control of vector-borne diseases and transmission risk. Based on WHO’s Phase 1 and 2 insecticide trials [38], malaria and dengue control is based on prevention of vector-host contact. Malaria preventive measures include the use of chemical insecticides, especially topical repellents, indoor residual spraying (IRS) and long-lasting insecticidal nets (LLINs). In Sai Yok District, IRS with residual dichloro-diphenyl-trichloroethane (DDT) was routinely used for several decades until it was replaced with deltamethrin in 2000, supplemented by ITNs and LLINs to-date [33, 37]. Community-based interventions (CBIs) are integrated with public health programs to tackle and control the expansion and emergence of dengue and vector-borne diseases (VBDs) in Sai Yok District and endemic areas. Residual malaria transmission is recognized as an issue for investigation and intervention [39, 40], but there is no consensus yet on how to quantify this concept [41]. Novel tools to interrupt residual transmission and complement core vector control tools are under development, such as improved outdoor adulticide spraying, outdoor adulticide delivery technology, volatile pyrethroids (VPs), mosquito traps, insecticide-treated barrier fencing, zooprophylaxis with systemic insecticides, lethal ovitraps, attract and kill solutions (e.g. attractive toxic sugar baits; oviposition lures; mating swarm lures), auto-dissemination of insecticides, larvicide delivery and insecticide treated net, cloth and blanket [42, 43].

### Study site

**Fig. 3:**
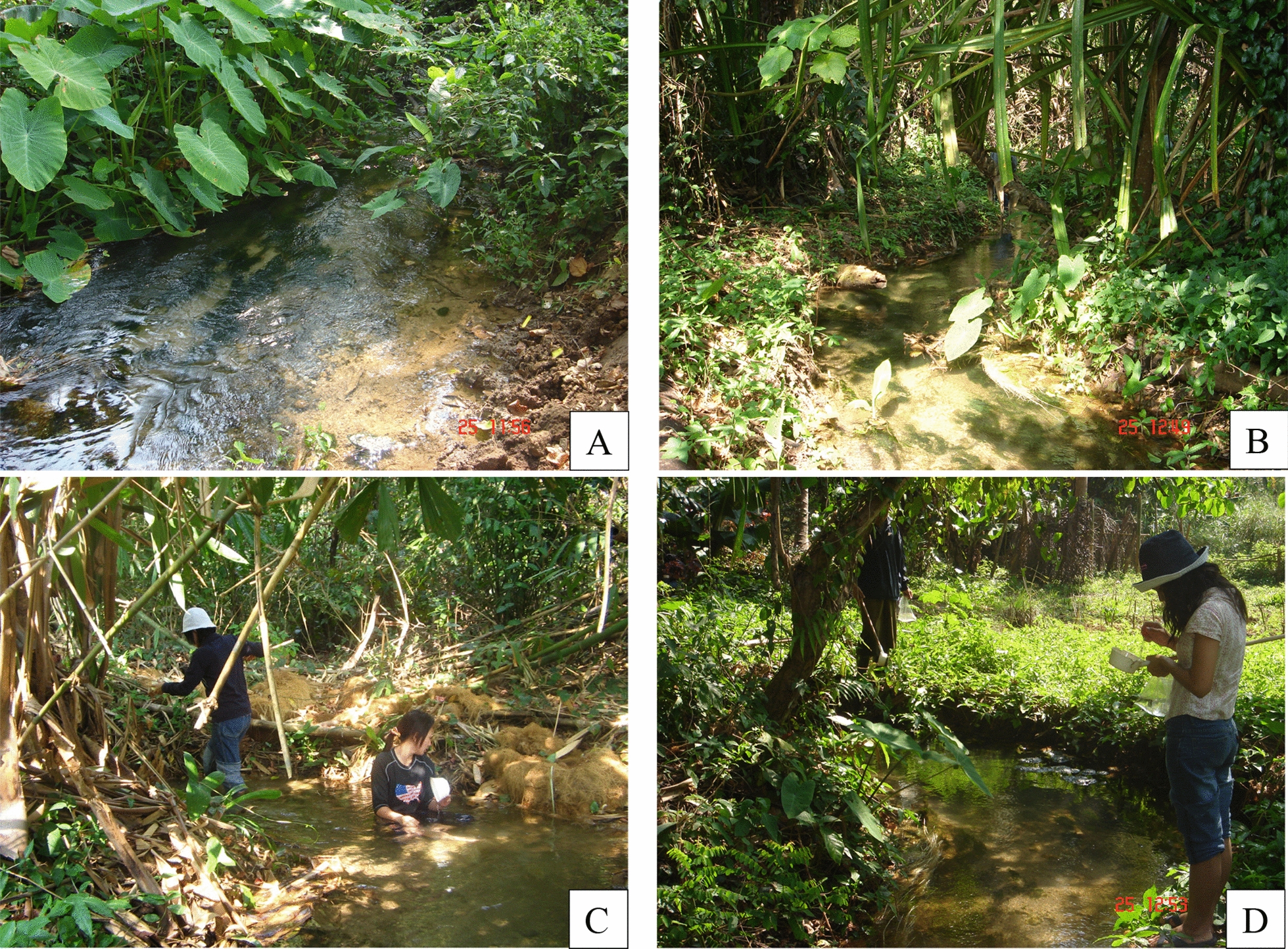
**A and B:** Mosquito larval habitats and surrounding in Pu Teuy study site and  **C and D:** *Anopheles* larval collecion

**Fig. 4:**
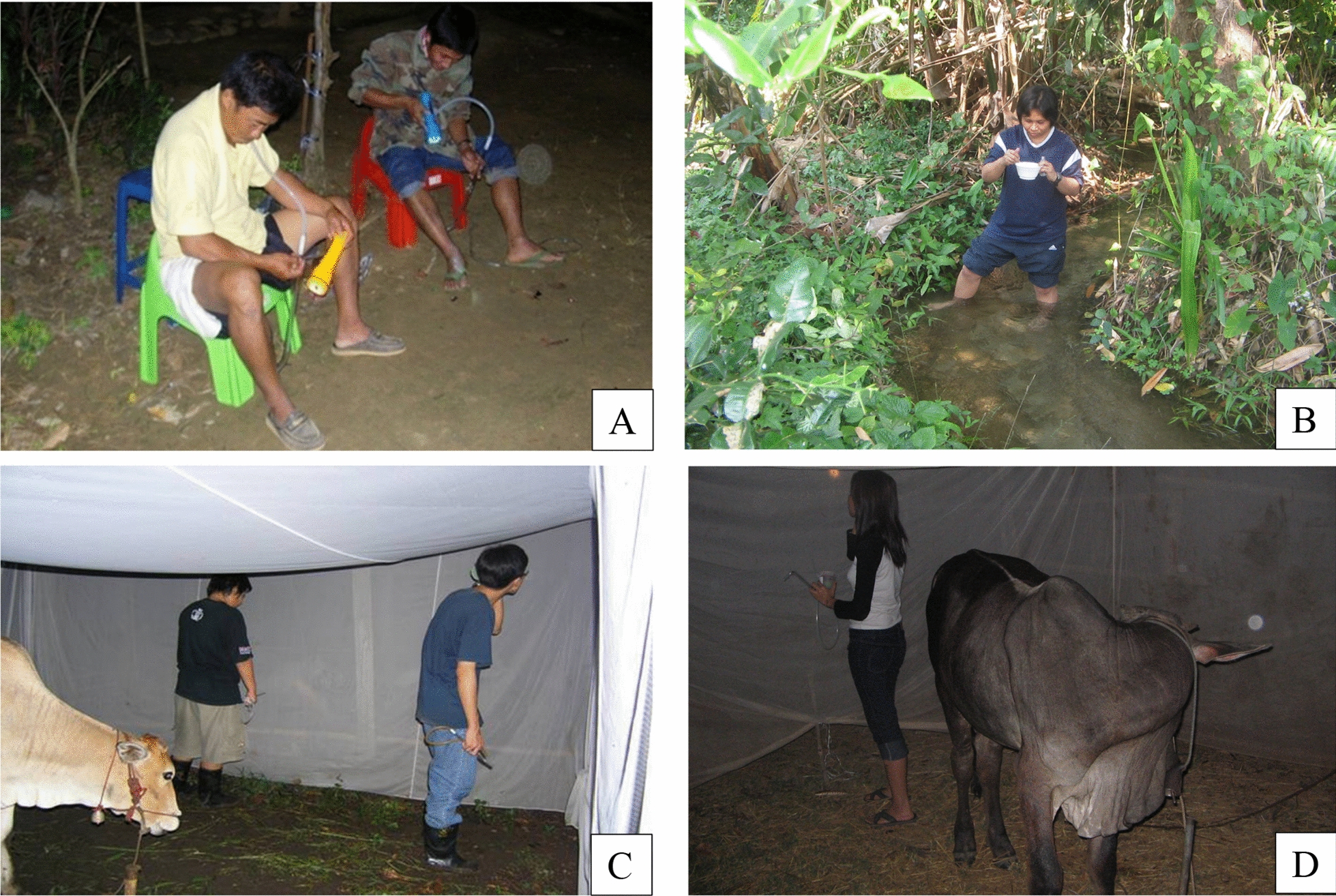
**A:** Human landing collections, **B:** *Anopheles* larval collection near Pu Teuy station, and **C** and **D:** cow-bait collections at Pu Tuey station Kanchanaburi Province

**Fig. 5:**
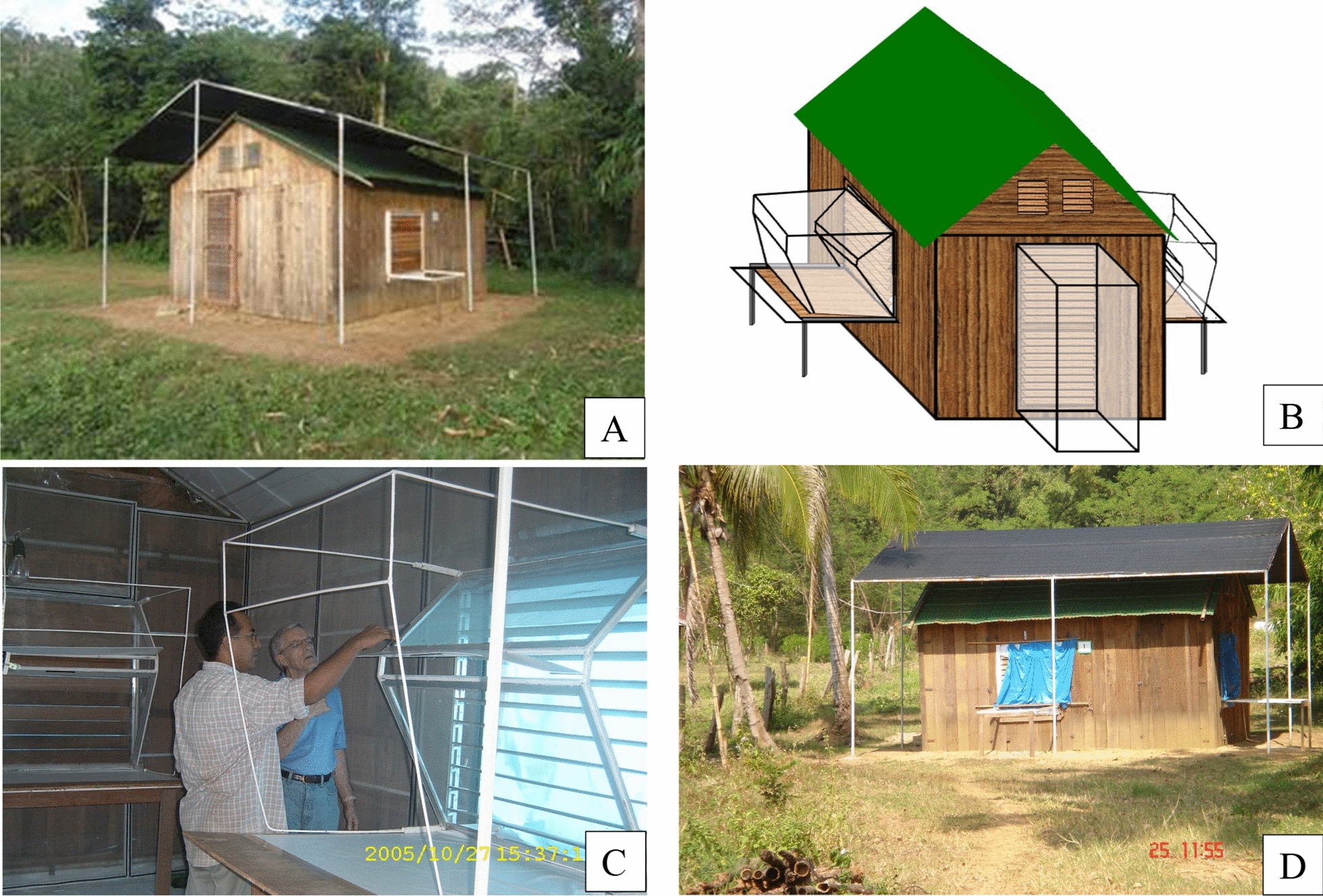
**A and D:** First experimental huts constructed to study mosquito responses to chemicals at the mosquito field research station (MFRS) in Pu Teuy; **B:** outside view and **C:** the inside view  showing the entrance traps on one of the hut openings [29]

The first mosquito study in Pu Teuy was conducted in 2001 [28, 49] given the favourable geography and landscape ecosystem for experimental mosquito vector research. The original field station comprised a 3-room temporary house and two experimental huts erected a few metres from the upper part of a stream that flows from the thick native forest surrounding the station, in the middle of Pu Teuy Village (Fig. 3). The huts were designed to assess efficacies of house-hold mosquito control interventions, such as insecticide-treated mosquito nets (ITNs) or indoor house spraying with residual insecticides (IRS), and provided information on the actual entry of mosquitoes, resting duration, indoor mortality or mortality after leaving the huts, and indoor feeding behaviours on human occupants. From 2007, the site facility was expanded to include a permanent insectary, an experimental room, a semi-field screened enclosure, and four experimental huts along with a resting station (Figs. 4 and 5) for conducting field experiments and trials. In this paper, the research activities carried out at the MFRS are divided into 2 main groups: (1) basic research studies on vector biology, taxonomy, genetics and toxicology, and 2) applied research studies focusing on response of mosquitoes to insecticides (repellents and non-repellents), using various methods ranging from diverse trapping systems, excito-repellency assay systems, semi-field screened enclosure and experimental huts fitted with window and door traps.

### Publications between 2001 and 2021

From 2001 to 2021, 51 publications linked to several research projects conducted at MFRS comprised of 13 (25.5%) basic scientific investigations on mosquito biology (10 publications), taxonomy (2 publications) and genetics (1 publication) (Table 1). In the applied science area, a total of 38 (74.5%) studies ranging from insecticide susceptibility (4 publications), excito-repellency assay systems (20 publications), high through-put test system (3 publications), and semi-field screened enclosure (3 publications), or experimental huts fitted with window and door traps (8 publications) were published (Table 1).

**Table 1:** Fifty one peer-reviewed publications by research categories conducted in Pu Teuy Village study site, Kanchanaburi Province between 2001 and 2021

Year	Research category	Number	Subject area	Publication
2003–2019	Basic Science	13		
2003			Biology	[28]
2005			Biology	[29]
2006			Biology	[78]
2006			Biology	[33]
2006			Taxonomy	[34]
2008			Biology	[79]
2008			Genetics	[31]
2009			Biology	[80]
2009			Biology	[81]
2009			Biology	[45]
2012			Biology	[52]
2019			Taxonomy	[73]
2021			Biology	[74]
2001–2020	Applied science	38	Vector Control	
2001			Excito-repellency system	[24]
2004			Excito-repellency system	[82]
2005			Excito-repellency system	[83]
2006			Excito-repellency system	[44]

Year	Research category	Number	Subject area	Publication
2007			High throughput screening system/experimental huts	[6]
2008			Excito-repellency system	[47]
2008			Insecticide/experimental huts	[63]
2008			Insecticide susceptibility	[84]
2009			Excito-repellency system	[85]
2009			Excito-repellency system	[86]
2009			Bottle assay & high-throughput screening system	[87]
2010			Insecticide/experimental huts	[88]
2010			High throughput screening system	[89]
2010			HLC/experimental huts	[64]
2011			Excito-repellency system	[90]
2011			Excito-repellency system	[70]
2011			Excito-repellency system	[69]
2012			BGS traps/ Semi-field	[56]
2012			Insecticide susceptibility	[91]
2012			Excito-repellency system	[92]
2012			Insecticide /experimental huts	[93]
2013			Excito-repellency system	[94]
2013			BGS-traps/ Semi-field	[23]
2013			Push–pull system/experimental huts	[95]

Year	Research category	Number	Subject area	Publication
2013			Insecticide/experimental huts	[96]
2014			Excito-repellency system	[97]
2014			Excito-repellency system	[98]
2014			Excito-repellency system	[99]
2014			Excito-repellency system	[100]
2014			Excito-repellency system	[101]
2015			Excito-repellency system	[102]
2016			Excito-repellency system	[25]
2017			BGS traps/ experimental huts	[103]
2018			BGS traps/ experimental huts	[65]
2018			Insecticide susceptibility	[104]
2019			Insecticide susceptibility	[105]
2020			Excito-repellency system	[43]
2021			Semi-field, outdoor	[46]

Peer reviewed published papers on *Aedes* and *Anopheles* species revealed 27 (53%) publications on *Aedes* spp., including 26 studies on *Aedes aegypti* and one on *Aedes albopictus* (Table 2)*.* A total of 24 (47%) publications focused on *Anopheles* spp., including *An. minimus* (7), *An. dirus* (5), *An. harrisoni* (3), *An. barbirostris* complex (2), and *Anopheles sawadwongporni* (1). Among these 24, six publications demonstrated the occurrence of sympatric species, including 2 on *An. maculatus* and *An. sawadwongporni* of the Maculatus Group [44, 45], 2 on *An. minimus* and *An. harrisoni* of the Minimus Complex [31, 46], one on *An. dirus* and *Anopheles baimaii* of the Dirus Complex [25], and one on the co-occurrence of *An. harrisoni* along with *Aedes aegypti* [47].

**Table 2:** Published papers on *Aedes* and *Anopheles* species in Pu Teuy Village, Kanchanaburi Province (2001–2021)

Mosquitoes	Number	Publications
*Aedes aegypti*	26	[6, 29, 64, 78, 81, 84–87, 89–91], [23, 56, 93–102], [65, 103]
*Aedes albopictus*	1	[79]
*Anopheles barbirostris* complex	2	[73, 74]
*Anopheles dirus*	5	[52, 80, 88, 92, 105]
*Anopheles harrisoni*	3	[43, 69, 70]
*Anopheles minimus*	7	[24, 28, 33, 34, 63, 83, 104]
*Anopheles sawadwongporni*	1	[82]
*Anopheles maculatus* & *Anopheles sawadwongporni*	2	[44, 45]
*Anopheles dirus* & *Anopheles baimaii*	1	[25]
*Anopheles minimus* & *Anopheles harrisoni*	2	[31, 46]
*Aedes aegypti* & *Anopheles harrisoni*	1	[47]

### The need for new vector control tools and the future role of insecticides

A better understanding of plasticity in host choice is critical for attributing disease reductions to the correct control mechanisms and is key to implementing the most effective malaria control strategy. This requires a study design for describing: how mosquito bites are distributed among different host species; how host choice is impacted by local host availability; and how this behaviour is impacted in the presence of different control measures (e.g., ITNs or IRS). Evaluating these parameters in different eco-epidemiological settings among vulnerable communities before and after mass distribution of novel vector control tools is an important focus of future work. Whilst host choice is not necessarily always dominated by extrinsic factors, it is important to determine how much this behaviour varies in different settings and what the key drivers are**.** Secondly, many of the dominant malaria vectors in Pu Tuey feed and rest outdoors, yet there is limited available protection against mosquito biting outdoors for at-risk populations [33, 40]. Pyrethroid resistance is on the rise in secondary vectors across the region, with increasing risk for emergence among dominant vectors [39]. Outdoor human activities in forests, including socializing during the evenings, delayed or late sleeping times and low bed net usage contribute to increased exposure to outdoor and indoor biting vectors [39]. Furthermore, all indoor control methods are ultimately unable to address residual transmission that occurs when people are inadequately protected by either IRS or LLINs [48, 49]. Residual transmission is likely to be holding back elimination in sub-districts and hotspots where core control tools have been deployed to scale whilst low levels of transmission stubbornly persist, year after year [37]. There is, therefore, an urgent need for new, more effective classes of vector control tools with different modes of action, as well as innovative strategies for their deployment to complement existing tools.

### Blood-feeding activity, host preference, and seasonal abundance studies of *Anopheles* mosquitoes

A good understanding of vector ecology, biology and behaviour of local *Anopheles* species is critically important to determine their responses to vector control and role in malaria transmission. This requires next generation vector surveillance tools to monitor behavioral responses, vectorial capacity, vector competence, comparative risk of pathogen transmission, which are needed in the design of locally derived and adapted vector control tools and strategies [50] (Fig. 4). Using HLC and CBC traps, Sungvornyothin et al. [33] reported the sympatric occurrence of molecularly-confirmed *An. minimus* s.s. and *An. harrisoni* in Pu Teuy, and preference of *An. harrisoni* to feed outdoor with a feeding peak at 19.00–20.00 h. However, the small number of *An. minimus* precluded a determination of its peak activity patterns. Overall, both species were more attracted to cattle than to humans ((*An. minimus:* 81.2%; *An. harrisoni*: 81.8%), more exophagic (*An. minimus*:15.8%; *An. harrisoni*: 15.4%) than endophagic (*An. minimus:* 2.9%; *An. harrisoni*:2.9%), irrespective of the season. Blood-feeding by *An. dirus* commenced immediately after sunset with a distinct peak of activity at 19.00–20.00 h and were more attracted to cattle than to humans, whereas *An. baimaii* females were equally attracted to both humans and cattle. Both species are sympatric and predominantly inhabit forest and forest-fringe [51]. This result was slightly different from Tananchai et al. [52] who found significantly greater numbers of *An. dirus* and *An. baimaii* collected from cattle baited traps as compared to human landing collections (P < 0.05), demonstrating that both species show a strong zoophilic behavior. The limitation of this ecological study was the explicit lack of the role of extrinsic factors, such as the influence of local host availability and indoor/outdoor trapping location, on host selection by *An. minimus* and *An. harrisoni* [33]*.*

Tananchai et al. [52] also documented the influence of rainfall on seasonal abundance of *Anopheles* mosquitoes and showed a positive correlation of adult densities with increased rainfall during July to August 2010 at Pu Teuy village. This supports previous observations of high rainfall supporting larval habitats for *An. dirus* that prefers temporary breeding ground habitats in Thailand [53], India [54] and Bangladesh [55]. An inverse relationship with rainfall for *An. minimus* [33] and *An. maculatus* was seen in the same locality [45] as these two species prefer breeding at the edges of slow-running streams [28, 33]. However, a negative association was found with a higher mean ambient temperature and relative humidity [51].

### Semi-field studies

Several semi-field system (SFS) experiments were conducted to evaluate the performance of BGS traps under varying mosquito population densities and the effects of spatial repellents on mosquito behaviours. In 2012, Salazar et al. [56] determined the feasibility of using the BG-Sentinel™ mosquito trap (BGS) as the pull component in a push–pull strategy to reduce indoor biting by *Aedes aegypti* at MFRS-SFS. Overall, the BGS trap was effective in recapturing three to five-day-old *Ae. aegypti* and recapture rates varied with BGS trap density and size of released mosquitoes. The highest cumulative percentage recapture over a 24 h period reached 98%, which is useful to guide the configuration and optimal trap numbers as part of a push–pull vector control strategy currently at the proof-of-concept stage of development in Thailand. Salazar et al. [23] measured BGS recapture rates of *Ae. aegypti* test cohorts that were exposed to either spatial repellent (SR) or control (chemical-free) treatments within SFS. Minimal and short-lived impacts (i.e., reduced attraction) on BGS trap catches following exposure to two volatile pyrethroids (VPs), transfluthrin (TFT) and metofluthrin), with no change in recapture densities on DDT as compared to matched controls. These findings suggest a combined SR and BGS approach is an effective push–pull strategy to reduce *Ae. aegypti* adults in and around homes.

In 2021, Sukkanon et al. [46] evaluated a SR prototype which is a passive emanator of airborne TFT for protecting humans against host-seeking mosquitoes. A plastic polyethylene terephthalate (PET) sheet (676 cm^2^) treated with 55 mg TFT (TFT-PET) was attached to the back of short-sleeve vest worn by a human mosquito collector in a semi-field and outdoor forested area. Field-caught, nonblood-fed female *An. minimus* were released in a 40 m length SFS where two collectors positioned at opposite ends conducted 12-h human-landing collections (HLC) over 30 replicates or nights. Although the protective efficacy of 67% between TFT-PET (intervention) and PET (untreated control) users was obtained in SFS, this level of protection was not replicated in outdoor setting where TFT-PET provided only 16% protection against *An. harrisoni* compared with an unprotected collector (*P* = 0.0213). The TFT-PET vest reduced non-anophelines landing by 1.4-fold compared with the PET control with a 29% protective efficacy. Given the diminished protective efficacy of TFT-PET in an open field environment, further research using different transfluthrin-treated formats is being planned.

During 2016–2020, studies were conducted on plant-based mosquito repellents [26, 57–59] in the SFS enclosure at MFRS. Evaluation of a binary mixture of β-caryophyllene (BCO) and an essential oil (EO) applied on two collectors positioned at the opposite end of the SFS showed that BCO-EO repellent provided a protective time against laboratory-reared *Aedes aegypti* bites for 4.7 h. Plant-based BCO-EO repellents may be more acceptable, practical and effective than contact insecticides for preventing outdoor biting mosquitoes but inferior than vapour-phase (spatial) repellents because they need not be applied to skin or clothing and may protect multiple occupants of spaces outside of treatable structures such as nets or houses [60].

### The need for innovative spatial repellents, treated clothing and their future role

Repellent technologies are important tools in the arsenal for preventing the spread of mosquito-borne diseases. Within this class, botanical and other biorational repellents [61] are diverse and are promising alternatives to synthetic pyrethroid spatial repellents, which are largely ineffective against pyrethroid-resistant mosquito vectors. Repellents target a wide variety of odorant receptors and physiological targets, suggesting that the potential for resistance to these chemistries is sufficiently low [62]. KU is currently conducting efficacy trials of spatial repellents in SFS for the Bite Interruption towards Elimination (BITE) Project sponsored by the Innovative Vector Control Consortium (IVCC). Current and future studies directed toward the development of long-lasting repellents could lead to promising alternatives to synthetic repellent formulations that are currently on the market.

### Experimental hut studies

Numerous studies that accurately measure the behavioural responses of indoor biting mosquitoes to insecticides using experimental huts were conducted during 2005 to 2018. In 2005, the first design of experimental hut (Fig. 5). measuring 4 m wide × 5 m long × 3.5 m high with three windows (1.125 × 1.175 m) and one door (0.8 × 2 m) affixed with entrance and exit traps (Fig. 4B and C) and constructed in the fashion of indigenous Thai homes was evaluated by Chareonviriyaphap et al. [29]. Assessment of the endophilic behaviour of *Aedes aegypti* showed a high degree of movement through the windows and doors in the huts with peaks of entry occurring at 08.40–10.40 h and 12.40–13.20 h, and peak of exit occurring at 16.40–17.40 h [29].

Baseline biting patterns of *Anopheles minimus* complex in experimental huts treated with DDT and deltamethrin showed peak activity of *An. minimus* females at 19:00–22:00 whereas post-treatment exposure showed greater landing activity during the first half of the evening [63]. In general, most of *An. minimus* females entered the hut treated with deltamethrin compared to DDT. The hut fitted with DDT-treated net panels showed a significant 71.5% decline in attempted blood feeding compared to 42.8% human-landing reduction in deltamethrin-treated panels (*P* < *0.005*) [63] suggesting excito-repellency or deterrence of DDT.

**Fig. 6:**
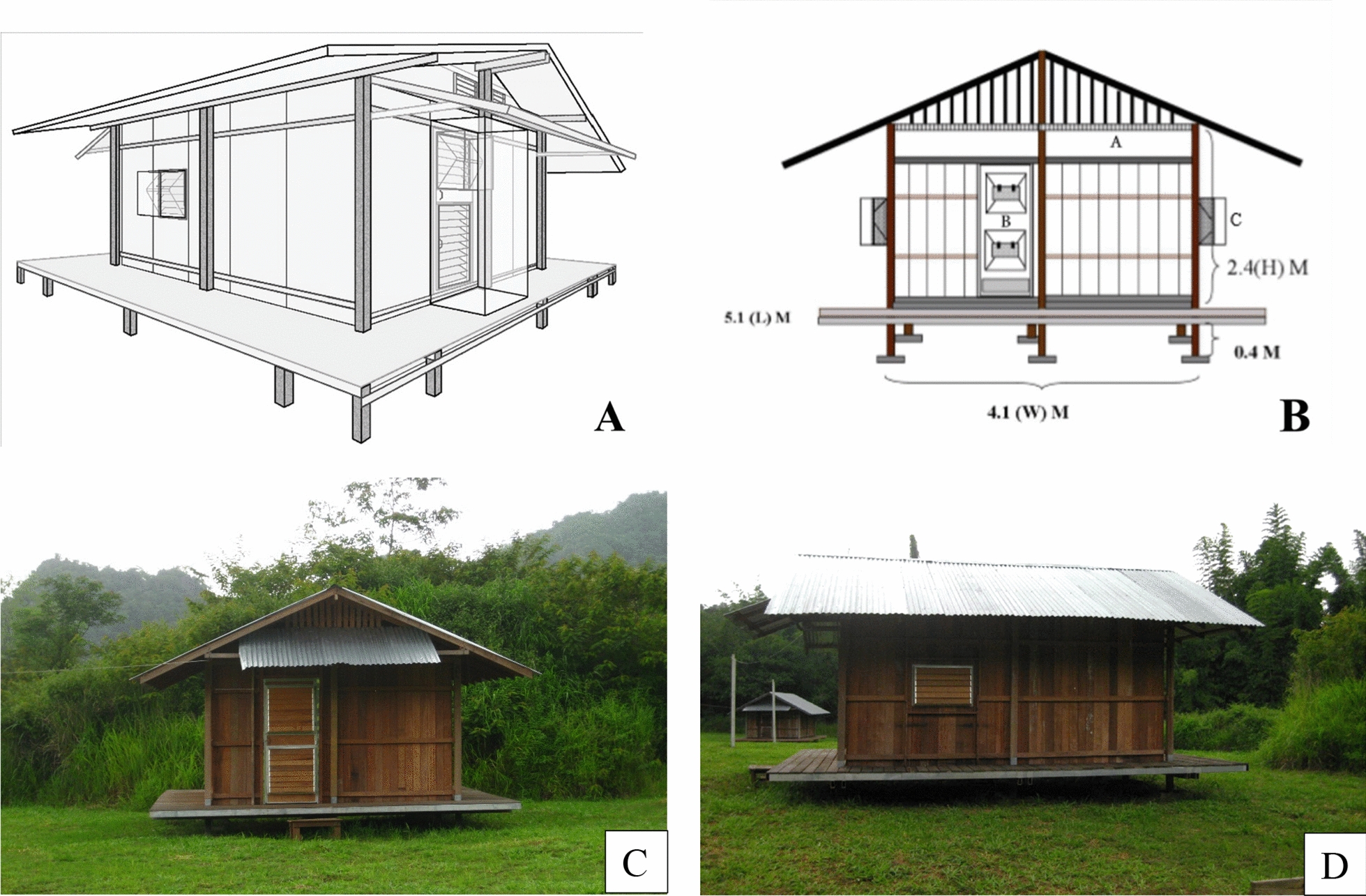
The improved experimental hut design used to study mosquito responses to chemical spatial repellency and push–pull concept for mosquito control in Pu Teuy study site, Kanchanaburi Province. **A** and **B:** the architectural design; **C** and **D:** outside view showing raised platform, perimeter walkway, exit and window traps [64]

**Fig. 7:**
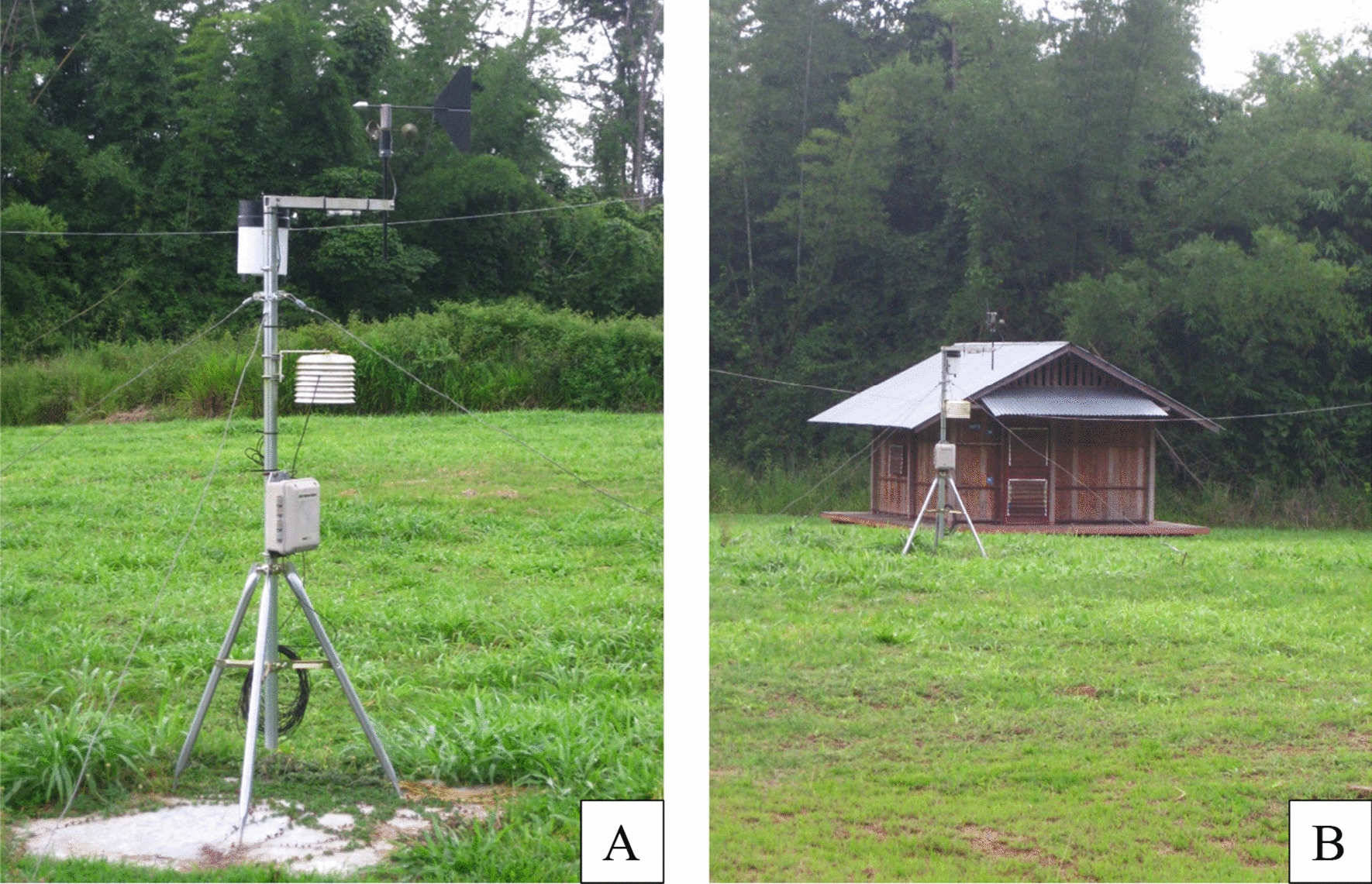
Weather station (**A**) close to an experimental hut (**B**), at the Mosquito Field Research Station (MFRS), Kanchanaburi Province

In 2010, Chareonviriyaphap et al. [64] made several modifications of the experimental huts which included: (1) a raised platform to prevent structural damage from termites and soil moisture; (2) cement ant traps placed underneath the raised platform to prevent predation of knock-down mosquitoes during chemical trials; (3) a walkway around the perimeter of the hut to facilitate mosquito removal from window and door traps (4) increased airflow between the ceiling and exterior roof to aid indoor heat dissipation and; 5 conducted weather station at MFRS (Figs. 6 and 7). A follow up evaluation of these unsprayed huts using field-reared *Ae. aegypti* mosquitoes validated the modification which served as a standard for studying mosquito entry and exit behaviours as part of the push–pull strategy of the research programme [59].

**Fig. 8:**
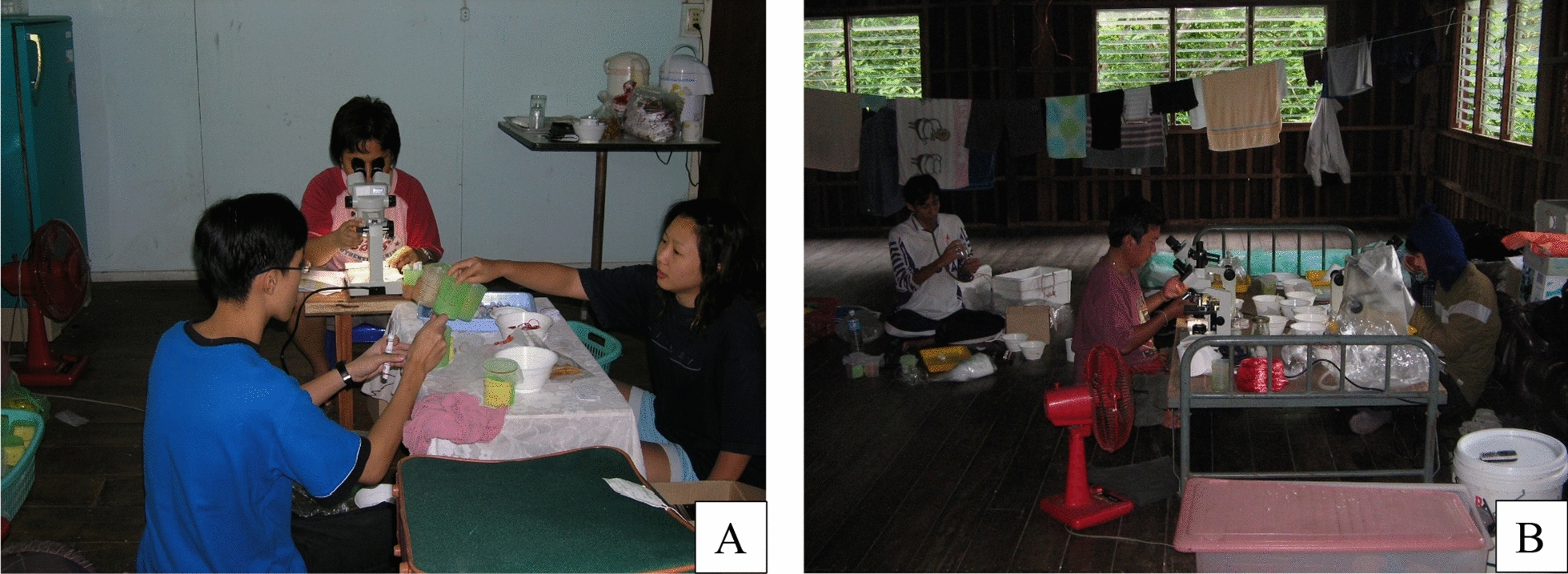
**A** and **B:** Mosquito species morphological identification in the field laboratory at Pu Teuy station, Kanchanaburi Province

In 2018, Salazar et al. [65] refined the ‘‘push–pull’’ strategy by varying the distance from human-occupied experimental huts for the placement of the ‘‘pull’’ component (BGS traps) to maximize the capture of mosquitoes. BGS traps were placed at portals of entry (windows or doors) or corners and at 0, 3 and 10 m from the huts. The location optimization trials revealed higher trap capture rates and reduction in entry of mosquitoes when the BGS traps were positioned nearer the experimental hut portals of entry than those placed in the corner of the huts [60].

In conclusion, experimental huts provide an improved system that can be used to realistically study the natural behaviour of wild free-flying populations of pathogen-transmitting mosquitoes and to evaluate the efficacy of various trap-based control strategies as well as LLINs and IRS. Their efficacy is enhanced by the addition of both eave and window traps thus making the design suitable for studying a wide range of mosquito entry and exit behaviours. The traps fitted onto the huts have eave baffles to control mosquito exit, which improve data reliability. Experiments with novel residual insecticides, concentrations, and formulations applied to the huts to assess the entomological impact of IRS will be the subject of future trials.

### Next generation residual insecticides and the future role of these tools

Malaria control relies primarily on insecticides through the use of LLINs complemented with IRS. One of the key elements in the Global Strategic Framework for Integrated Vector Management [66] is ensuring that there is “adequate, evidence-based guidance on combining IRS with LLINs and other malaria control interventions.” Programmatic decisions rely on evidence of impact of combining IRS and active case detection (ACD)/ passive case detection (PCD) in low transmission areas of Thailand with high rates of LLIN access and pyrethroid-resistant vectors. As such, the need for new products and novel approaches to malaria vector control has been widely acknowledged as a global health priority. Future plans of KU include the assessment of new IRS products with novel active ingredients for public health, e.g., third-generation IRS (NgenIRS) products that are defined as IRS products effective at killing pyrethroid-resistant mosquitoes and which have target duration of residual efficacy lasting at least 6 months. As these new products need to be applied in endemic communities, KU will adopt an integrated vector management (IVM)strategy through: (1) strengthening inter- and intra-sectoral action and collaboration; (2) engaging and mobilizing communities; (3) enhancing vector surveillance and monitoring and evaluation of interventions; and (4) scaling up and integrating tools and approaches [66]. Activities within these four pillars complement one another and are consistent with the WHO’s Global Vector Control Response 2017–2030 promoting community engagement and mobilization to achieve effective and locally adapted vector control and enhance protective behaviours among the population [67, 68].

### Genomic and molecular studies of mosquitoes from Pu Teuy mosquito field research station

In Thailand, seven malaria vector species, *An. baimaii*, *An. dirus*, *An. minimus*, *Anopheles aconitus*, *An. maculatus*, *Anopheles pseudowillmori,* and *An. sawadwongporni* are represented by three species complexes or groups. Species complexes or group comprise morphologically indistinguishable sibling species which are invariably characterized by inter- and intraspecific variation in vector competence, vectorial capacity, insecticide susceptibility and host-seeking behaviours (Fig. 8). Precise identification of anopheline mosquitoes is essential for a better understanding of their potential role in malaria transmission and improving the effectiveness of vector control strategies [51]. Molecular polymerase chain reaction (PCR) assays are the current “gold standard” and recently used for sibling species identification [33]. A multiplex allele-specific polymerase chain reaction (AS-PCR) successfully identified two sibling species of *An. minimus* (former species A) and *An. harrisoni* (former species C) at MFRS [64].

Subsequently, Sungvornyothin et al. [34] compared the reliability of both morphological characters and molecular PCR to differentiate two sibling species of the *An. minimus* complex*,* which are characterized by the presence of a presector pale spot on the wing costa of *An. minimus* or both presector and humeral pale spot for *An. harrisoni*. Spatial and temporal variations of wing scale pattern render these two morphological characters unreliable for the precise identification of *An. minimus* and *An. harrisoni*. However, molecular PCR identification reliably discriminated between these 2 sibling species. Poolprasert et al. [31] compared isozyme frequencies of seven *An. minimus* populations from collections in MFRS and four malaria endemic districts in Kanchanaburi Province using starch gel electrophoresis. Gene flow analysis showed that *An. minimus* and *An. harrisoni* from Pu Teuy were phylogenetically grouped closely in the same cluster. As sibling species have different host seeking behaviours and vector competence, morphological and molecular techniques are often used *a priori* to verify their species identity and to tailor species-specific control strategies. In 2011, Malaithong et al. [69] and Tisgratog et al. [70] used the AS-PCR restriction fragment length polymorphism (RFLP) techniques, respectively, for identification of *An. harrisoni* populations from Pu Tuey prior to performing the excito-repellency assays (ERA) of insecticides. RFLP-PCR used enzyme addition after the DNA amplification, which gives species identification after sequence analysis of the PCR products without designing specific primers, whereas AS-PCR has the advantage of being species-specific and provides a rapid diagnosis without the sequencing step [71, 72]. In 2012, Tananchai et al. [52] used the AS-PCR technique to confirm the species identity of exophagic *An. dirus* and *An. baimaii* in Pu Teuy Village. Prior to conducting ERAs of mosquitoes exposed to pyrethroids, AS-PCR technique accurately confirmed the identity of field-caught *An. harrisoni* and *An. dirus* from Pu Teuy Village [25]. In 2019, Brosseau et al. [73] developed a multiplex AS-PCR technique to identify five species of the *An. barbirostris* group, with some specimens from Pu Teuy. AS-PCR used species-specific differences within the sequences of the internal transcribed spacer 2 (ITS2), a ribosomal DNA gene (rDNA) widely used to differentiate cryptic species of Asian Anopheles complexes [44] and reliably identified five species in the Barbirostris Complex in Thailand [73]. Another recent study by Udom et al. [74] on the same group included PCR-identified Pu Teuy specimens and provided a national map of Anopheles species complex distribution. From these genetic studies in Pu Teuy, correct species identification is essential and mandatory for basic and applied mosquito studies and for the evaluation of vector control strategies [34]. As population genetics, genetically modified mosquitoes and gene drive are promising tools for malaria control, molecular genetic studies will bridge the gap between the laboratory and the field to support malaria vector control.

### Research capacity building

Internationalized higher education system is about “bringing the Thai education system to the international standard as well as making Thailand to be the country of higher education destination for Southeast Asia” according to Kirtikara [75]. The National Scheme of Education 2017–2036 has prioritized the development of research and innovative workforce to enhance the national competitiveness. The Ministry of Higher Education, Science, Research and Innovation’s policy of “Reinventing the University System" aims to eliminate the weaknesses of Thai higher education institutions and improve their quality to international standards in response to rapid global change and both predictable and unpredictable risks such as rapid technological development, social change and others. In adopting this policy, KU has a mandate to ensure the development of appropriate strategies for global and frontier research; technology development and innovation; area based and community development; and professional development and moral and intellectual cultivation. In this context, the utilisation of MFRS by of postgraduate students will support a new economic model aimed at pulling Thailand out of the ‘middle-income trap’, and push the country into the high-income bracket, collectively known as “Thailand 4.0”. Previous and recent activities at the MFRS have emphasized and explored an orphaned area of research that could have high impact on the control of vector-borne diseases and pests of humans and animals of economic importance. The facilities at the MFRS also support the institutional capacity building to strengthen international-level research capacities by providing technical support from various global and local partners including private industry.

KU has succeeded in renewed funding for two key research support programmes: firstly, the Thailand Science Research and Innovation (TSRI) awarded by KU and Ministry of Education; and secondly, the Asia Pacific Malaria Elimination Network (APMEN)//Asia Pacific Leaders Malaria Alliance Secretariat (APMLA) to support regional training courses and fellowships. By 2021, a total of 23 postgraduate students (7 Master and 16 Doctoral level) at the Department of Entomology, Faculty of Agriculture, completed their studies with a focus on mosquito biology, taxonomy, genetics, and mosquito control (Table 3). In addition, another 36 overseas students and research staff from various countries also completed their field studies at MFRS (Table 4). APMEN supported an international training course on malaria vector surveillance for elimination (MVSE) for programme and research entomologists in 19 countries from the Asia–Pacific region. Course curriculum comprised vector identification and vector surveillance methods were conducted on 29 Sept to 12 Oct 2019, and field training on new mosquito surveillance methods on 6 to 10 Sept 2018. These programmes comprised an optimal and balanced mix of lectures, laboratory training and field practicals at KU in Bangkok and MFRS in Kanchanaburi Province. A team of international experts, collaborated with staff and students from the Department of Entomology, KU, and Malaria Consortium, co-designed and delivered the courses. While the emphasis of the courses was directed at deepening the knowledge of participants regarding the diversity, biology and identification of the main vector species complexes in the Asia–Pacific region, much time was also spent on collection and processing of specimens, data gathering methodology, geographic information system applications, insecticide susceptibility tests, insectary establishment and maintenance of mosquito colonies. Feedback from participants and collaborators suggest that the MVSE courses were valuable and contributed to a range of specialized technical and analytical skills relevant to vectors and malaria elimination in the Asia–Pacific region. Benefits included standardization of methods, improved capacity to apply or adapt similar techniques to combat the rising tide of arbovirus threats in the region, such as dengue, chikungunya, Zika and others [76]. These training programmes help to advance knowledge and understanding of vector biology and control to the participants, as illustrated by a feedback from an international participant:

**Table 3:** Number of Kasetsart University graduate students conducted their studies at Pu Teuy Mosquito Vector Research Station, Kanchanaburi Province between 2006 and 2021

Level	Year of graduation	Number	Nationality
M.Sc	2006	1	Thai
	2008	1	Thai
	2009	1	Thai
	2011	2	Thai
	2012	2	Thai
Ph.D	2006	1	Thai
	2007	1	Thai
	2008	1	Thai
	2011	1	Thai
	2012	2	Thai, Philippines
	2013	1	Thai
	2015	2	Thai
	2017	1	Thai
	2018	1	Thai
	2020	1	Thai
	2021	4	Thai, Korean, Cambodia*, Uganda*
Total		23	

^*^On-going Ph.Ds

**Table 4:** Overseas students and researchers who conducted their studies at Pu Teuy Mosquito Vector Research Station, Kanchanaburi Province between 2009 and 2019

Year	University/Institute	Country	Number
2009	Eijkman Institute for Molecule Biology	Indonesia	1
2009	Uniformed Services University	USA	1
2010	Uniformed Services University	USA	3
2011	University of Montpellier	France	1
2011	Research Institute for Tropical Medicine	Philippines	2
2012	Norwegian university of Life Sciences	Norway	2
2013	French Research Institute for Sustainable Development	France	4
2013	University of Montpellier	France	1
2013	University of Health Sciences	Cambodia	1
2014	University of Florida	USA	2
2015	University of Notre Dame	USA	2
2016	University of Notre Dame	USA	1
2016	National Chung Hsing University	Taiwan	4
2017	University of Notre Dame	USA	2
2017	Universiti Sains Malaysia	Malaysia	2
2018	Anti-Malaria Campaign Headquarters	Sri Lanka	1
2018	National Chung Hsing University	Taiwan	4
2018	University of Oxford	England	1
2018	University of Liverpool	England	1
2018	London School of Hygiene and Tropical Medicine	England	1
2019	University of Montpellier	France	1
	Total		37

*‘Entomologists play a key role in the national malaria programme. Having specifically trained at MVSE Training Programme, it allows the entomologists within the National Department of Health as well as in our research arm a step forward for PNG as this will highlight the interest and substantiate the importance of the entomological information produced in the vector surveillance movement towards vector-borne disease control and elimination in my country.*’’ Ms. Naomi Vincent, Vector Borne Disease Surveillance Officer, National Department of Health, Papua New Guinea [77].

## Conclusion

MFRS in Pu Teuy is a unique and well-organized research facility geared to elucidate various aspects of the biology, surveillance and ecology of mosquito vector species, with the aim of developing and evaluating innovative new tools and cost-effective technologies relevant to vector control. Evidence-based information from previous studies were shared with the national malaria and dengue control programmes to support decision-making and assessment of current strategies. This is essential to ensure that scarce resources are efficiently used for maximum impact in order to assist MOPH in developing public health policy on disease vector control, strategic planning and implementation.

Over the past 20 years, the MFRS benefited from international and constructive partnerships with the University of Montpellier (France), University of Notre Dame (USA), University of Florida (USA), Eijkman Institute for Molecular Biology (Indonesia), Uniformed Services University of the Health Sciences (USA), French Research Institute for Sustainable Development (IRD, France), University of Oxford (England), University of Liverpool (England), London School of Hygiene and Tropical Medicine (England), University of Health Sciences (Cambodia), Research Institute for Tropical Medicine (Philippines), Norwegian University of Life Sciences (Norway), Universiti Sains Malaysia (Malaysia), National Chung Hsing University (Taiwan), Anti Malaria Campaign Headquarters (Sri Lanka), Greenwich University (UK), and James Cook University (Australia), resulting in 37 overseas student and researcher exchanges. MFRS has had many successful research collaborations, resulting in an interactive coalition that is committed to supporting KU research policy without jeopardizing its integrity. This coalition also involves policy-makers from the MOPH and Food and Drug Administration in Thailand. Networking with partners from other Thai universities have also been established. The MFRS coalition supports research and training, but also assists the process of transferring research findings into policy. MFRS also provides additional support to address the technical and knowledge gaps identified provided by the network, with the intent for these to be further presented and discussed with MOPH. A focus on targeted research, knowledge and information exchange, and improved programme management will guide the MFRS moving forward. A coalition of partners has a mutual benefit for the national and international medical research communities moving towards globalization, and enriching human resource and talents.

Future studies for the development of long-lasting botanical or bio-rational repellents are planned as these could lead to promising alternatives to current repellent products that are safer to humans and the environment. Other important control technologies include alternative IRS products or third-generation IRS, chemical dose optimization for operational programmes, controlled release system for prolonging the protective efficacy, advances for effective management of natural physical variables and environmental conditions (e.g., temperature, wind velocity), and user-friendly devices for personal protection. Additional studies coupled with human behaviour observations are needed to assess the impact on outdoor-biting malaria vectors in Thailand, involving primary and secondary malaria vectors as well as important arboviral vectors under both semi-field and natural field conditions. Furthermore, improved novel mosquito traps such as lethal ovitraps, oviposition lures, mating swarm lures and improved insecticide treated nets, clothing and blanket are of significant interest. The results obtained from this research station site will contribute to achieving the national malaria elimination goal by 2024.

## Data Availability

Not applicable.

## Abbreviations

ACD: Active case detection
API: Annual parasite incidence
APMEN: Asia Pacific Malaria Elimination Network
AS-PCR: Allele-specific polymerase chain reaction
BGS: BG-Sentinel™ trap
BITE: Bite Interruption Towards Elimination
CBIs: Community-based interventions
rDNA: Ribosomal DNA gene
ER: Excito-repellency assays
DDT: Dichlorodiphenyltrichloroethane
DF: Dengue fever
DVBD: Division of Vector-Borne Diseases
HLC: Human-landing collections
HPH: Health Promotion Hospitals
IRS: Indoor residual spraying
ITS2: Internal transcribed spacer 2
IVM: Integrated vector management
IVCC: Innovative Vector Control Consortium
JE: Japanese encephalitis
KU: Kasetsart University
LLINs: Long-lasting insecticide treated bed nets
MFRS: Mosquito Field Research Station
MOPH: Ministry of Public Health
MVSE: Malaria Vector Surveillance for Elimination
NgenIRS: Third-generation IRS
PCD: Passive case detection
PCR: Polymerase chain reaction
PCR–RFLP: Polymerase chain reaction-restriction fragment length polymorphism
PET: Polyethylene terephthalate
PHO: Provincial Health Office
SAO: Subdistrict Administrative Organization
SFS: Semi-field screened enclosure
SR: Spatial repellent
TFT: Transfluthrin
TSRI: Thailand Science Research and Innovation
VBDs: Vector-borne diseases
WHO: World Health Organization
VP: Volatile pyrethroids

## References

[1] Andreazza F, Oliveira EE, Martins GF (2021). Implications of sublethal insecticide exposure and the development of resistance on mosquito physiology, behavior, and pathogen transmission. Insects.

[2] WHO. Report on antimalarial drug efficacy, resistance and response: 10 years of surveillance (2010–2019). Geneva: World Health Organization; 2020.

[3] Bowman LR, Donegan S, McCall PJ. Is dengue vector control deficient in effectiveness or evidence?: Systematic review and meta-analysis. PLoS Negl Trop Dis. 2016;10:e0004551. 10.1371/journal.pntd.0004551 PMC4795802 26986468

[4] Horstick O, Boyce R, Runge-Ranzinger S (2018). Building the evidence base for dengue vector control: searching for certainty in an uncertain world. Pathog Global Health.

[5] Ferguson HM, Ng'habi KR, Walder T, Kadungula D, Moore SJ, Lyimo I (2008). Establishment of a large semi-field system for experimental study of African malaria vector ecology and control in Tanzania. Malar J.

[6] Grieco JP, Achee NL, Chareonviriyaphap T, Suwonkerd W, Chauhan K, Sardelis MR, et al. A new classification system for the actions of IRS chemicals traditionally used for malaria control. PLoS One. 2007;2:e716. 10.1371/journal.pone.0000716 PMC1934935 17684562

[7] Ismail I, Notananda V, Schepens J (1975). Studies on malaria and responses of *Anopheles balabacencis* and *Anopheles minimus* to DDT spraying in Thailand. Part II Post-spraying observations Acta Trop.

[8] Chareonviriyaphap T, Bangs MJ, Suwonkerd W, Kongmee M, Corbel V, Ngoen-Klan R (2013). Review of insecticide resistance and behavioral avoidance of vectors of human diseases in Thailand. Parasit Vectors.

[9] Knols BG, Njiru BN, Mathenge EM, Mukabana WR, Beier JC, Killeen GF (2002). MalariaSphere: a greenhouse-enclosed simulation of a natural *Anopheles gambiae* (Diptera: Culicidae) ecosystem in western Kenya. Malar J.

[10] Helinski ME, Hassan MM, El-Motasim WM, Malcolm CA, Knols BG, El-Sayed B (2008). Towards a sterile insect technique field release of *Anopheles arabiensis *mosquitoes in Sudan: irradiation, transportation, and field cage experimentation. Malar J.

[11] Ritchie SA, Johnson PH, Freeman AJ, Odell RG, Graham N, Dejong PA, et al. A secure semi-field system for the study of *Aedes aegypti*. PLoS Negl Trop Dis. 2011;5:e988. 10.1371/journal.pntd.0000988 PMC3062535 21445333

[12] Denz A, Njoroge MM, Tambwe MM, Champagne C, Okumu F, van Loon JJA (2021). Predicting the impact of outdoor vector control interventions on malaria transmission intensity from semi-field studies. Parasit Vectors.

[13] DVBD. Guide to malaria elimination for Thailand’s local administrative organizations and the health network. Division of Vector Borne Diseases, Thailand.; 2019.

[14] Ogoma SB, Lorenz LM, Ngonyani H, Sangusangu R, Kitumbukile M, Kilalangongono M (2014). An experimental hut study to quantify the effect of DDT and airborne pyrethroids on entomological parameters of malaria transmission. Malar J.

[15] Mamai W, Simard F, Couret D, Ouédraogo GA, Renault D, Dabiré KR (2016). Monitoring dry season persistence of *Anopheles gambiae* sl populations in a contained semi-field system in southwestern Burkina Faso. West Africa J Med Entomol.

[16] Niang A, Nignan C, Serge Poda B, Sawadogo SP, Roch Dabire K, Gnankine O (2019). Semi-field and indoor setups to study malaria mosquito swarming behavior. Parasit Vectors.

[17] Stevenson JC, Simubali L, Mudenda T, Cardol E, Bernier UR, Vazquez AA (2018). Controlled release spatial repellent devices (CRDs) as novel tools against malaria transmission: a semi-field study in Macha. Zambia Malar J.

[18] Tambwe MM, Moore SJ, Chilumba H, Swai JK, Moore JD, Stica C (2020). Semi-field evaluation of freestanding transfluthrin passive emanators and the BG sentinel trap as a "push-pull control strategy" against *Aedes aegypti* mosquitoes. Parasit Vectors.

[19] Okech BA, Gouagna LC, Walczak E, Kabiru EW, Beier JC, Yan G (2004). The development of *Plasmodium falciparum* in experimentally infected *Anopheles gambiae* (Diptera: Culicidae) under ambient microhabitat temperature in western Kenya. Acta Trop.

[20] WHO. Guidelines for laboratory and field testing of long-lasting insecticidal mosquito nets. Geneva: World Health Organization; 2005.

[21] WHO. Guidelines for testing mosquito adulticides for indoor residual spraying and treatment of mosquito nets. Geneva: World Health Organization; 2006.

[22] Aldersley A, Pongsiri A, Bunmee K, Kijchalao U, Chittham W, Fansiri T (2019). Too "sexy" for the field? Paired measures of laboratory and semi-field performance highlight variability in the apparent mating fitness of *Aedes aegypti* transgenic strains. Parasit Vectors.

[23] Salazar FV, Achee NL, Grieco JP, Prabaripai A, Ojo TA, Eisen L (2013). Effect of *Aedes aegypti* exposure to spatial repellent chemicals on BG-Sentinel trap catches. Parasit Vectors.

[24] Chareonviriyaphap T, Sungvornyothin S, Ratanatham S, Prabaripai A (2001). Insecticide-induced behavioral responses of *Anopheles minimus*, a malaria vector in Thailand. J Am Mosq Control Assoc.

[25] Tainchum K, Nararak J, Boonyuan W, Bangs MJ, Chareonviriyaphap T (2016). Behavioral responses of *Anopheles* species (Culicidae: Diptera) with varying surface exposure to pyrethroid-treated netting in an excito-repellency test system. J Vector Ecol.

[26] Nararak J. Efficacy and persistence of promising repellents towards green vector control programs. PhD thesis; Kasetsart University (Thailand) and University of Montpellier (France). 2020.

[27] SAO. Directorate of Subdistrict Administrative Organizations of Ta Sao. [Internet]. 2021 Available from: http://www.thasao-kan.go.th/about. Accessed 2 June 2021.

[28] Chareonviriyaphap T, Prabaripai A, Bangs MJ, Aum-Aung B (2003). Seasonal abundance and blood feeding activity of *Anopheles minimus* Theobald (Diptera: Culicidae) in Thailand. J Med Entomol.

[29] Chareonviriyaphap T, Suwonkerd W, Mongkalangoon P, Achee N, Grieco J, Farlow B (2005). The use of an experimental hut for evaluating the entering and exiting behavior of *Aedes aegypti* (Diptera : Culicidae), a primary vector of dengue in Thailand. J Vector Ecol.

[30] Tainchum K, Kongmee M, Manguin S, Bangs MJ, Chareonviriyaphap T (2015). *Anopheles* species diversity and distribution of the malaria vectors of Thailand. Trends Parasitol.

[31] Poolprasert P, Manguin S, Bangs MJ, Sukhontabhirom S, Poolsomboon S, Akaratanakul P (2008). Genetic structure and gene flow of *Anopheles minimus* and *Anopheles harrisoni* in Kanchanaburi Province. Thailand J Vector Ecol.

[32] Rwegoshora RT, Sharpe RG, Baisley KJ, Kittayapong P (2002). Biting behavior and seasonal variation in the abundance of *Anopheles minimus* species A and C in Thailand. Southeast Asian J Trop Med Public Health.

[33] Sungvornyothin S, Muenvorn V, Garros C, Manguin S, Prabaripai A, Bangs MJ (2006). Trophic behavior and biting activity of the two sibling species of the *Anopheles minimus* complex in western Thailand. J Vector Ecol.

[34] Sungvornyothin S, Garros C, Chareonviriyaphap T, Manguin S (2006). How reliable is the humeral pale spot for identification of cryptic species of the *Minimus* Complex?. J Am Mosq Control Assoc.

[35] Tananchai C, Manguin S, Bangs MJ, Chareonviriyaphap T (2019). Malaria vectors and species complexes in Thailand: implications for vector control. Trends Parasitol.

[36] Meankaew P, Kaewkungwal J, Khamsiriwatchara A, Khunthong P, Singhasivanon P, Satimai W (2010). Application of mobile-technology for disease and treatment monitoring of malaria in the "Better Border Healthcare Programme". Malar J.

[37] DVBD. Division of Vector Borne Diseases.Thailand [Internet]. 2021. Available from: https://drive.google.com/drive/folders/1D5qQj_9LLV4NIYtQSSxhbmF2BGkaIOXp. Accessed 2 Feb 2021.

[38] WHO. Guidelines for malaria vector control. Geneva, World Health Organization; 2019. 30844152

[39] Edwards HM, Sriwichai P, Kirabittir K, Prachumsri J, Chavez IF, Hii J (2019). Transmission risk beyond the village: entomological and human factors contributing to residual malaria transmission in an area approaching malaria elimination on the Thailand-Myanmar border. Malar J.

[40] Carnevale P, Manguin S (2021). Review of issues on residual malaria transmission. J Infect Dis.

[41] Hii J, Hustedt J, Bangs MJ (2021). Residual malaria transmission in select countries of Asia-Pacific region: old wine in a new barrel. J Infect Dis.

[42] Okumu FO, Govella NJ, Moore SJ, Chitnis N, Killeen GF. Potential benefits, limitations and target product-profiles of odor-baited mosquito traps for malaria control in Africa. PLoS One. 2010;5:e11573. 10.1371/journal.pone.0011573 PMC2904375 20644731

[43] Sukkanon C, Nararak J, Bangs MJ, Hii J, Chareonviriyaphap T. Behavioral responses to transfluthrin by *Aedes aegypti*, *Anopheles minimus*, *Anopheles harrisoni*, and *Anopheles dirus* (Diptera: Culicidae). PLoS One. 2020;15:e0237353. 10.1371/journal.pone.0237353 PMC7423148 32785255

[44] Muenworn V, Akaratanakul P, Bangs MJ, Parbaripai A, Chareonviriyaphap T (2006). Insecticide-induced behavioral responses in two populations of *Anopheles maculatus* and *Anopheles sawadwongporni*, malaria vectors in Thailand. J Am Mosq Control Assoc.

[45] Muenworn V, Sungvornyothin S, Kongmee M, Polsomboon S, Bangs MJ, Akrathanakul P (2009). Biting activity and host preference of the malaria vectors *Anopheles maculatus* and *Anopheles sawadwongporni* (Diptera: Culicidae) in Thailand. J Vector Ecol.

[46] Sukkanon C, Tisgratog R, Muenworn V, Bangs MJ, Hii J, Chareonviriyaphap T (2021). Field evaluation of a spatial repellent emanation vest for personal protection against outdoor biting mosquitoes. J Med Entomol.

[47] Polsomboon S, Grieco JP, Achee NL, Chauhan KR, Tanasinchayakul S, Pothikasikorn J (2008). Behavioral responses of catnip (*Nepeta cataria*) by two species of mosquitoes, *Aedes aegypti* and *Anopheles harrisoni*, in Thailand. J Am Mosq Control Assoc.

[48] Killeen GF (2014). Characterizing, controlling and eliminating residual malaria transmission. Malar J.

[49] Durnez L, Coosemans M. Residual transmission of malaria: an old issue for new approaches. In: *Anopheles* mosquitoes-new insights into malaria vectors Manguin S, Ed. InTech Open; 2013. 671–704.

[50] Farlow R, Russell TL, Burkot TR (2020). Nextgen Vector Surveillance Tools: sensitive, specific, cost-effective and epidemiologically relevant. Malar J.

[51] Manguin S, Garros C, Dusfour I, Harbach RE, Coosemans M (2008). Bionomics, taxonomy, and distribution of the major malaria vector taxa of *Anopheles* subgenus Cellia in Southeast Asia: an updated review. Infect Genet Evol.

[52] Tananchai C, Tisgratog R, Juntarajumnong W, Grieco JP, Manguin S, Prabaripai A (2012). Species diversity and biting activity of *Anopheles dirus* and *Anopheles baimaii* (Diptera: Culicidae) in a malaria prone area of western Thailand. Parasit Vectors.

[53] Baimai V, Kijchalao U, Sawadwongporn P, Green CA (1988). Geographic distribution and biting behaviour of four species of the *Anopheles dirus* complex (Diptera: Culicidae) in Thailand. Southeast Asian J Trop Med Publ Health.

[54] Dutta P, Khan SA, Bhattarcharyya DR, Khan AM, Sharma CK, Mahanta J (2010). Studies on the breeding habitats of the vector mosquito *Anopheles baimai* and its relationship to malaria incidence in Northeastern region of India. EcoHealth.

[55] Rosenberg R, Maheswary N. Forest malaria in Bangladesh. II. Transmission by *Anopheles dirus*. Am J Trop Med Hyg. 1982;31:183–91. 10.4269/ajtmh.1982.31.183 7072883

[56] Salazar FV, Achee NL, Grieco JP, Prabaripai A, Eisen L, Shah P (2012). Evaluation of a peridomestic mosquito trap for integration into an *Aedes aegypti* (Diptera: Culicidae) push-pull control strategy. J Vector Ecol.

[57] Nararak J, Sathantriphop S, Kongmee M, Mahiou-Leddet V, Ollivier E, Manguin S, et al. Excito-repellent activity of beta-caryophyllene oxide against *Aedes aegypti* and *Anopheles minimus*. Acta Trop. 2019;197:105030. 10.1016/j.actatropica.2019.05.021 31121148

[58] Nararak J, Di Giorgio C, Sukkanon C, Mahiou-Leddet V, Ollivier E, Manguin S, et al. Excito-repellency and biological safety of β-caryophyllene oxide against *Aedes albopictus* and *Anopheles dirus* (Diptera: Culicidae). Acta Trop. 2020;210:105556. 10.1016/j.actatropica.2020.105556 32485168

[59] Tisgratog R, Sanguanpong U, Grieco JP, Ngoen-Kluan R, Chareonviriyaphap T (2016). Plants traditionally used as mosquito repellents and the implication for their use in vector control. Acta Trop.

[60] Killeen GF, Moore SJ (2012). Target product profiles for protecting against outdoor malaria transmission. Malar J.

[61] Strickman D. Putting the rational into biorational. In: Advances in the biorational control of medical and veterinary pests. ACS Publications 2018. 3–6.

[62] Norris EJ, Coats JR (2017). Current and future repellent technologies: The potential of spatial repellents and their place in mosquito-borne disease control. Int J Environ Res Public Health.

[63] Polsomboon S, Poolprasert P, Suwonkerd W, Bangs MJ, Tanasinchayakul S, Akratanakul P (2008). Biting patterns of *Anopheles minimus* complex (Diptera: Culicidae) in experimental huts treated with DDT and deltamethrin. J Vector Ecol.

[64] Chareonviriyaphap T, Grieco JP, Suwonkerd W, Prabaripai A, Polsomboon S, Thainchum K (2010). An improved experimental hut design for the study of *Aedes aegypti* (Diptera: Culicidae) movement patterns in Thailand. J Vector Ecol.

[65] Salazar FV, Chareonviriyaphap T, Grieco JP, Eisen L, Prabaripai A, Ojo TA (2018). Influence of location and distance of biogents sentinel traps from human-occupied experimental huts on *Aedes aegypti* recapture and entry into huts. J Am Mosq Control Assoc.

[66] WHO. Global strategic framework for integrated vector management. Geneva, World Health Organization; 2004.

[67] WHO. Global Vector Control Response 2017–2030. Geneva, World Health Organization; 2017.

[68] Pengvanich V (2011). Family leader empowerment program using participatory learning process for dengue vector control. J Med Assoc Thai.

[69] Malaithong N, Tisgratog R, Tainchum K, Prabaripai A, Juntarajumnong W, Bangs MJ (2011). Locomotor behavioral responses of *Anopheles minimus* and *Anopheles harrisoni* to alpha-cypermethrin in Thailand. J Am Mosq Control Assoc.

[70] Tisgratog R, Tananchai C, Bangs MJ, Tainchum K, Juntarajumnong W, Prabaripai A (2011). Chemically induced behavioral responses in *Anopheles minimus* and *Anopheles harrisoni* in Thailand. J Vector Ecol.

[71] Lubis NZ, Muis K, Nasution LH (2018). Polymerase chain reaction-restriction fragment length polymorphism as a confirmatory test for onychomycosis. Open Access Maced J Med Sci.

[72] Xu R, Ogino S, Lip V, Fang H, Wu BL (2003). Comparison of PCR-RFLP with allele-specific PCR in genetic testing for spinal muscular atrophy. Genet Test.

[73] Brosseau L, Udom C, Sukkanon C, Chareonviriyaphap T, Bangs MJ, Saeung A (2019). A multiplex PCR assay for the identification of five species of the *Anopheles barbirostris* complex in Thailand. Parasit Vectors.

[74] Udom C, Thanispong K, Manguin S, Chareonviriyaphap T, Fungfuang W (2021). Trophic Behavior and Species Diversity of the *Anopheles barbirostris* complex (Diptera: Culicidae) in Thailand. J Med Entomol.

[75] Kirtikara K. Higher education in Thailand and the national reform roadmap. Invited Paper presented at the Thai-US Education Roundtable. 2001;9:

[76] APMEN. Training report: 2nd international course on malaria vector surveillance for elimination (MVSE). 2019.

[77] APMEN. International training course on malaria vector surveillance for elimination (MVSE). [Internet]. 2019. Available from: https://orene.org/wp-content/uploads/2019/11/MVSE-Brief.pdf. Accessed 8 August 2021.

[78] Suwonkerd W, Mongkalangoon P, Parbaripai A, Grieco J, Achee N, Roberts D (2006). The effect of host type on movement patterns of *Aedes aegypti* (Diptera : Culicidae) into and out of experimental huts in Thailand. J Vector Ecol.

[79] Pothikasikorn J, Bangs MJ, Boonplueang R, Chareonviriyaphap T (2008). Susceptibility of various mosquitoes of Thailand to nocturnal subperiodic *Wuchereria bancrofti*. J Vector Ecol.

[80] Sungvornyothin S, Kongmee M, Muenvorn V, Polsomboon S, Bangs MJ, Prabaripai A (2009). Seasonal abundance and bloodfeeding activity of *Anopheles dirus sensu lato* in western Thailand. J Am Mosq Control Assoc.

[81] Suwannachote N, Grieco JP, Achee NL, Suwonkerd W, Wongtong S, Chareonviriyaphap T (2009). Effects of environmental conditions on the movement patterns of *Aedes aegypti* (Diptera: Culicidae) into and out of experimental huts in Thailand. J Vector Ecol.

[82] Chareonviriyaphap T, Prabaripai A, Bangs MJ (2004). Excito-repellency of deltamethrin on the malaria vectors, *Anopheles minimus*, *Anopheles dirus*, *Anopheles swadiwongporni*, and *Anopheles maculatus*, in Thailand. J Am Mosq Control Assoc.

[83] Potikasikorn J, Chareonviriyaphap T, Bangs MJ, Prabaripai A (2005). Behavioral responses to DDT and pyrethroids between *Anopheles minimus* species A and C, malaria vectors in Thailand. Am J Trop Med Hyg.

[84] Thanispong K, Satfiantriphop S, Chareonviriyaphap T (2008). Insecticide resistance of *Aedes aegypti* and *Culex quinquefasciatus* in Thailand. J Pest Sci.

[85] Thanispong K, Achee NL, Bangs MJ, Grieco JP, Suwonkerd W, Prabaripai A (2009). Irritancy and repellency behavioral responses of three strains of *Aedes aegypti* exposed to DDT and alpha-cypermethrin. J Med Entomol.

[86] Mongkalangoon P, Grieco JP, Achee NL, Suwonkerd W, Chareonviriyaphap T (2009). Irritability and repellency of synthetic pyrethroids on an *Aedes aegypti* population from Thailand. J Vector Ecol.

[87] Dusfour I, Achee NL, Sardelis MR, Chareonviriyaphap T, Grieco JP (2009). Comparison of a novel high-throughput screening system with the bottle assay for evaluating insecticide toxicity. J Pestic Sci.

[88] Malaithong N, Polsomboon S, Poolprasert P, Parbaripai A, Bangs MJ, Suwonkerd W (2010). Human-landing patterns of *Anopheles dirus* sensu lato (Diptera: Culicidae) in experimental huts treated with DDT or deltamethrin. J Med Entomol.

[89] Thanispong K, Achee NL, Grieco JP, Bangs MJ, Suwonkerd W, Prabaripai A (2010). A high throughput screening system for determining the three actions of insecticides against *Aedes aegypti* (Diptera: Culicidae) populations in Thailand. J Med Entomol.

[90] Boonyuan W, Kongmee M, Bangs MJ, Prabaripai A, Chareonviriyaphap T. Host feeding responses of *Aedes aegypti* (L.) exposed to deltamethrin. J Vector Ecol. 2011;36:361–72. 10.1111/j.1948-7134.2011.00177.x 22129408

[91] Pimnon S, Juntarajumnong W, Thanispong K, Chareonviriyaphap T. Diagnostic doses of two pyrethroids currently used for control of *Aedes aegypti* L. (Diptera: Culicidae), a vector of dengue. Agr Nat Resour. 2012;46:538–45.

[92] Tananchai C, Tisgratog R, Grieco JP, Chareonviriyaphap T (2012). Pyrethroid induced behavioral responses of *Anopheles dirus*, a vector of malaria in Thailand. J Vector Ecol.

[93] Achee N, Masuoka P, Smith P, Martin N, Chareonviryiphap T, Polsomboon S (2012). Identifying the effective concentration for spatial repellency of the dengue vector *Aedes aegypti*. Parasit Vectors.

[94] Suwansirisilp K, Visetson S, Prabaripai A, Tanasinchayakul S, Grieco JP, Bangs MJ (2013). Behavioral responses of *Aedes aegypti* and *Culex quinquefasciatus* (Diptera: Culicidae) to four essential oils in Thailand. J Pest Sci.

[95] Tainchum K, Polsomboon S, Grieco JP, Suwonkerd W, Prabaripai A, Sungvornyothin S (2013). Comparison of *Aedes aegypti* (Diptera: Culicidae) resting behavior on two fabric types under consideration for insecticide treatment in a push-pull strategy. J Med Entomol.

[96] Manda H, Shah P, Polsomboon S, Chareonviriyaphap T, Castro-Llanos F, Morrison A, et al. Contact irritant responses of *Aedes aegypti* using sublethal concentration and focal application of pyrethroid chemicals. PLoS Negl Trop Dis. 2013;7:e2074. 10.1371/journal.pntd.0002074 PMC3585116 23469302

[97] Noosidum A, Chareonviriyaphap T, Chandrapatya A. Synergistic repellent and irritant effect of combined essential oils on *Aedes aegypti* (L.) mosquitoes. J Vector Ecol. 2014;39:298–305. 10.1111/jvec.12104 25424258

[98] Sathantriphop S, Thanispong K, Sanguanpong U, Achee NL, Bangs MJ, Chareonviriyaphap T (2014). Comparative behavioral responses of pyrethroid-susceptible and -resistant *Aedes aegypti* (Diptera: Culicidae) populations to citronella and eucalyptus oils. J Med Entomol.

[99] Boonyuan W, Grieco JP, Bangs MJ, Prabaripai A, Tantakom S, Chareonviriyaphap T. Excito-repellency of essential oils against an *Aedes aegypti* (L.) field population in Thailand. J Vector Ecol. 2014;39:112–22. 10.1111/j.1948-7134.2014.12077.x 24820563

[100] Tainchum K, Ritthison W, Sathantriphop S, Tanasilchayakul S, Manguin S, Bangs MJ (2014). Influence of time of assay on behavioral responses of laboratory and field populations *Aedes aegypti* and *Culex quinquefasciatus* (Diptera: Culicidae) to DEET. J Med Entomol.

[101] Polsomboon S, Poolprasert P, Bangs MJ, Suwonkerd W, Grieco JP, Achee NL (2014). Effects of physiological conditioning on behavioral avoidance by using a single age group of *Aedes aegypti* exposed to deltamethrin and DDT. J Med Entomol.

[102] Sathantriphop S, Kongmee M, Tainchum K, Suwansirisilp K, Sanguanpong U, Bangs MJ (2015). Comparison of field and laboratory-based tests for behavioral response of *Aedes aegypti* (Diptera: Culicidae) to repellents. J Econ Entomol.

[103] Salazar FV, Chareonviriyaphap T, Grieco JP, Prabaripai A, Polsomboon S, Gimutao KA (2017). BG-sentinel trap efficacy as a component of proof-of-concept for push-pull control strategy for dengue vector mosquitoes. J Am Mosq Control Assoc.

[104] Thanispong K, Sathantriphop S, Tisgratog R, Tainchum K, Sukkanon C, Bangs MJ (2018). Optimal discriminating concentrations of six synthetic pyrethroids for monitoring insecticide susceptibility in *Anopheles minimus* (Diptera: Culicidae), a primary malaria vector in Thailand. J Econ Entomol.

[105] Sukkanon C, Bangs MJ, Nararak J, Hii J, Chareonviriyaphap T (2019). Discriminating lethal concentrations for transfluthrin, a volatile pyrethroid compound for mosquito control in Thailand. J Am Mosq Control Assoc.

