# Correction: Scientific achievements and reflections after 20 years of vector biology and control research at the Pu Teuy mosquito field research station, Thailand

**DOI:** 10.1186/s12936-022-04426-w

**Published:** 2023-01-23

**Authors:** Patcharawan Sirisopa, Chutipong Sukkanon, Michael J. Bangs, Sutkhet Nakasathien, Jeffrey Hii, John P. Grieco, Nicole L. Achee, Sylvie Manguin, Theeraphap Chareonviriyaphap

**Affiliations:** 1 Department of Entomology, Faculty of Agriculture, Kasetsart University, Bangkok, 10900 Thailand; 2 Department of Medical Technology, School of Allied Health Sciences, Walailak University, Nakhon Si Thammarat, 80160 Thailand; 3 Department of Agronomy, Faculty of Agriculture, Kasetsart University, Bangkok, 10900 Thailand; 4 College of Public Health, Medical and Veterinary Sciences, James Cook University, North Queensland, QLD 4810 Australia; 5 Department of Biological Sciences, Eck Institute for Global Health, University of Notre Dame, Notre Dame, IN USA; 6 HSM, Univ. Montpellier, CNRS, IRD, IMT, Montpellier, France

**Correction: Malaria Journal (2022) 21:44** https://doi.org/10.1186/s12936-022-04061-5

Following publication of the original article [1], it came to the authors' attention that the following funding was not acknowledged in the funding declaration: the International Research Network (IRN), Thailand Research Fund Organization (Grant No: IRN58W0003).

The funding declaration has now been corrected in the published article to acknowledge this funding source. The authors thank you for reading this correction and apologize for any inconvenience caused.

## References

[1] Sirisopa P, Sukkanon C, Bangs MJ, Nakasathien S, Hii J, Grieco JP, Achee NL, Manguin S, Chareonviriyaphap T (2022). Scientific achievements and reflections after 20 years of vector biology and control research at the Pu Teuy mosquito field research station, Thailand. Malar J.

